# Targeting necroptosis as therapeutic potential in chronic myocardial infarction

**DOI:** 10.1186/s12929-021-00722-w

**Published:** 2021-04-09

**Authors:** Chanon Piamsiri, Chayodom Maneechote, Natthaphat Siri-Angkul, Siriporn C. Chattipakorn, Nipon Chattipakorn

**Affiliations:** 1 Cardiac Electrophysiology Research and Training Center, Faculty of Medicine, Chiang Mai University, Chiang Mai, 50200 Thailand; 2 Cardiac Electrophysiology Unit, Department of Physiology, Faculty of Medicine, Chiang Mai University, Chiang Mai, 50200 Thailand; 3 Center of Excellence in Cardiac Electrophysiology Research, Chiang Mai University, Chiang Mai, 50200 Thailand

**Keywords:** Chronic myocardial infarction, Heart failure, Necroptosis, Cell death pathways

## Abstract

Cardiovascular diseases (CVDs) are considered the predominant cause of morbidity and mortality globally. Of these, myocardial infarction (MI) is the most common cause of CVD mortality. MI is a life-threatening condition which occurs when coronary perfusion is interrupted leading to cardiomyocyte death. Subsequent to MI, consequences include adverse cardiac remodeling and cardiac dysfunction mainly contribute to the development of heart failure (HF). It has been shown that loss of functional cardiomyocytes in MI-induced HF are associated with several cell death pathways, in particular necroptosis. Although the entire mechanism underlying necroptosis in MI progression is still not widely recognized, some recent studies have reported beneficial effects of necroptosis inhibitors on cell viability and cardiac function in chronic MI models. Therefore, extensive investigation into the necroptosis signaling pathway is indicated for further study. This article comprehensively reviews the context of the underlying mechanisms of necroptosis in chronic MI-induced HF in in vitro, in vivo and clinical studies. These findings could inform ways of developing novel therapeutic strategies to improve the clinical outcomes in MI patients from this point forward.

**Supplementary Information:**

The online version contains supplementary material available at 10.1186/s12929-021-00722-w.

## Background

Cardiovascular diseases (CVDs) are the world’s most important causes of morbidity and mortality, with 110.6 million cases contributing to over 8.9 million deaths globally [1]. Myocardial infarction (MI) is the most prevalent of the CVD categories [2]. MI is defined as myocardial cell death due to significant and sustained ischemia [3]. Patients with a history of MI have been shown to be the group with the highest risk of impaired cardiac function and the development of heart failure (HF) [4, 5]. HF has been recognized as a major determinant of adverse prognosis after MI [6, 7]. There is accumulating evidence to demonstrate that HF greatly increases mortality risk during both the acute and chronic phases of MI [8]. Acute MI results from acute obstruction of the coronary arteries, leading to myocardial ischemia [9, 10]. Oxidative stress along with the inflammatory response plays a critical role in the acute phase of MI [9, 10]. Under ischemic conditions, excessive accumulation of reactive oxygen species (ROS) induces DNA damage and cytochrome C release from mitochondria, leading to intrinsic apoptosis [11–13]. Likewise, an inflammatory process during the acute phase of MI, primarily mediated by tumor necrosis factor-α (TNF-α), also triggers cardiomyocyte apoptosis via the extrinsic pathway [11–13]. In addition, cardiomyocyte apoptosis can be triggered by several neurohormonal activation including adrenaline, noradrenaline and angiotensin II [14–16]. In contrast, chronic MI is a term widely used in the literature to refer to the protracted pathophysiological processes following the ischemic insult which is characterized by cardiac fibrosis and cardiac remodeling [17, 18]. Indeed, the exact time frame of the chronic phase of MI is still inconsistently defined [19], but it is essentially characterized by cardiac remodeling and subsiding of inflammation [17, 18]. During this phase, the alterations in LV architecture including chamber dilatation, cardiomyocyte hypertrophy, scar maturation, and increased wall stiffness are observed, and all of which could lead to LV dysfunction and heart failure [9, 18]. Different types of cell death mechanisms occur throughout the disease progression, both in the acute and chronic phases.

**Fig. 1:**
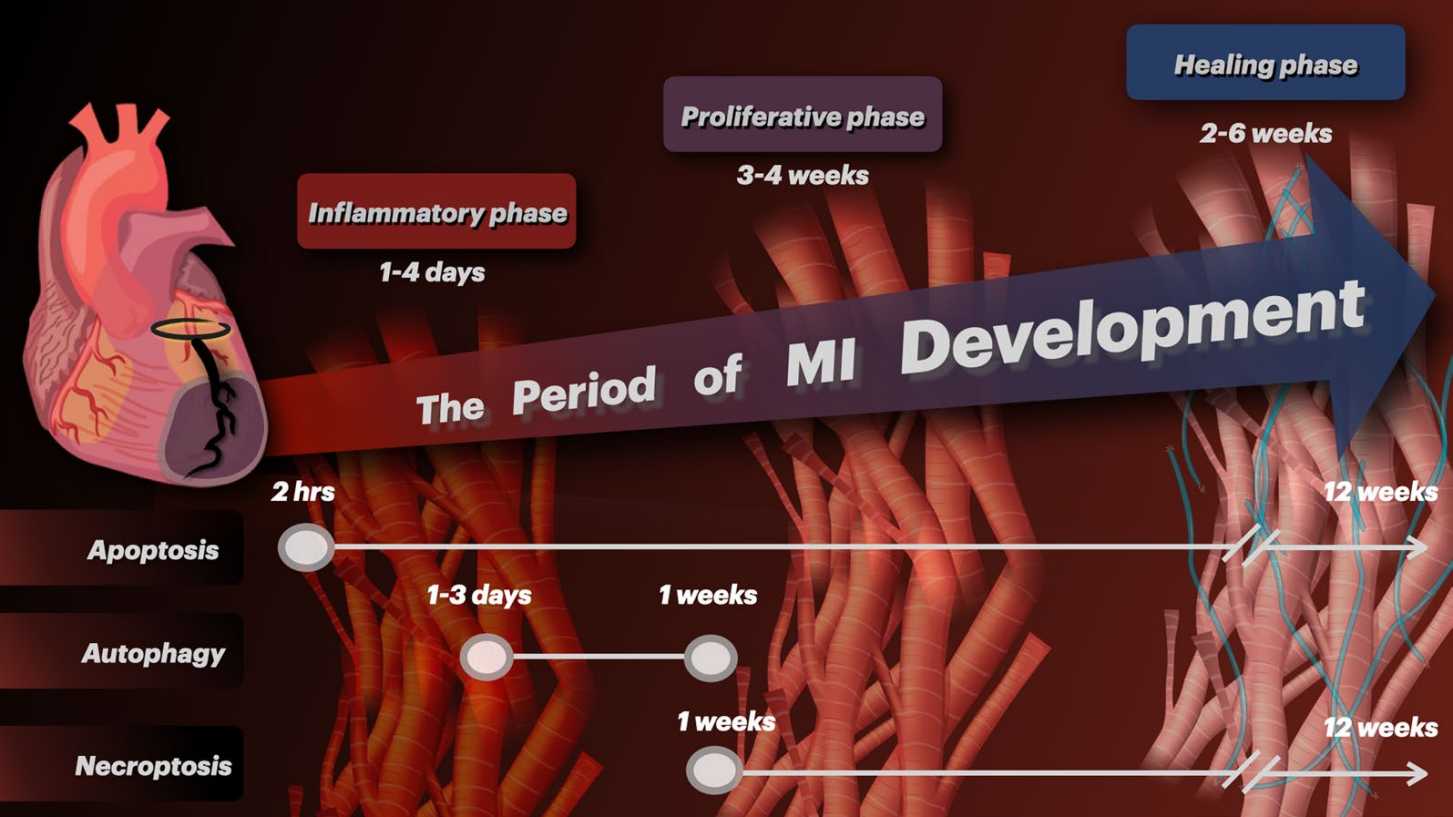
The chronological change of MI progression. The progression of MI following myocardial ischemia involves three phases. The inflammatory phase occurs 1 to 4 days after the myocardium becomes ischemic. The proliferative phase then follows and lasts for 3 to 4 weeks after MI. Lastly, the myocardium become repaired and remodeled in the healing phase at 2 to 6 weeks of MI progression. Different cell death mechanisms occur throughout the disease progression. Cardiomyocyte apoptosis could be demonstrated at as early as 2 h following MI and remains detectable up to 12 weeks. The autophagy machinery is upregulated within 1 to 3 days after MI in the rat models. At 1 week following MI, the autophagic flux becomes impaired and necroptosis emerges. Cardiomyocyte necroptosis is persistently increased up to 12 weeks in an experimental model. The progressive death of cardiomyocyte is responsible for deterioration of cardiac contractile function

Cardiomyocyte apoptosis is detectable at 2 h up to 12 weeks of MI progression [11, 12, 20]. The recent study by Zhang et al. (2020) demonstrated that autophagy machinery was intensely upregulated approximately at 1 to 3 days after MI in rats [21]. Afterward, the autophagy flux process became impaired at 1 week following MI [21]. They also found that the necroptosis markers (i.e. receptor-interacting serine/threonine-protein kinase 1; RIPK1 and receptor-interacting serine/threonine-protein kinase 3; RIPK3) were persistently increased up to 12 weeks after LAD ligation [21]. Taken together, current evidence indicates that apoptosis is the most profound mechanism of cardiomyocyte death in the earliest period of MI. At about the same time, the autophagy machinery is upregulated to degrade and recycle damaged proteins and organelles in order to support cellular metabolism. However, when the ischemia is prolonged, the autophagy flux becomes impaired and could no longer efficiently support cell survival, thereby leading to induction of necroptosis. This information is illustrated in Fig. 1. It is important to point out that the time course of multiple cell death mechanisms may overlap to a considerable degree. Time-course experiments should be conducted in the future to clarify the interplay among multiple cell death pathways in the chronic MI models.

Myocardial cell death is a fundamental process in both physiological and pathological conditions, the process is controlled by several signaling pathways including apoptosis, necroptosis and ferroptosis [21–24]. Once cardiomyocyte loss has occurred, the surviving cardiomyocytes in the non-infarcted area are subjected to an increased pressure and/or volume load and become hypertrophic [21]. This compensatory response could become maladaptive in long term, leading to the death of the remnant cardiomyocytes in the peri-infarction tissue [24–27]. As illustrated in the Fig. 1, both apoptosis and necroptosis contribute to the death of cardiomyocytes in chronic remodeling [12, 26, 27]. This notion is supported by the study of Palojoki et al. (2001), which demonstrated that the progressive ventricular wall distension due to increased ventricular filling pressure after MI could further induce cardiomyocyte apoptosis in the non-infarcted myocardium [12]. In addition, apart from apoptosis, the study of Lichý et al. (2019) showed that the non-infarcted myocardial tissue exhibited increased phosphorylated receptor-interacting protein kinase 3 (p-RIPK3), suggesting that necroptosis also play a role in pathological remodeling after MI [25]. Therefore, the combination of interventions targeting multiple cell death mechanisms is an interesting candidate for further investigation on cardioprotective strategies against MI.

Although these cell death pathways have been previously investigated, the relative impact of these pathways in the context of chronic MI is currently unclear. Cardiomyocyte loss during MI leads to the replacement of damaged tissue with a fibrotic scar produced by fibroblasts and myofibroblasts [28]. Either cardiomyocyte death or cardiac fibrosis induces cardiac geometrical and biomechanical changes as evidenced by the impairment of systolic and diastolic functions after MI [28]. Previous studies have shown that increased necroptosis mediators are not only related to the loss of cardiomyocytes, but also associated with more severe cardiac remodeling and poorer cardiac contractile parameters over the period of chronic MI development [29–34]. Therefore, characterizing of the contribution of necroptosis in the development of MI may pave the way for the devising of novel strategies for therapeutic interventions to improve the clinical outcomes in MI patients.

Although various form of cell death mechanisms (i.e. necroptosis, apoptosis and autophagy) contribute to the loss of cardiomyocytes in chronic MI, the precise mechanisms or mediators which ultimately determine the dominant cell death pathway remains elusive. Comparing to necroptosis, the involvement of apoptosis and autophagy in MI has been more extensively investigated. Autophagy is a fundamental catabolic process involving the degradation of damaged proteins and organelles [35–37]. The appropriate degrees of autophagy are essential for cellular homeostasis as well as adaptive responses to stress [21, 35, 38–40]. In the early stage of ischemia, autophagic process is initiated by the increased levels of beclin1 and LC3 II/I [35, 37]. Upon prolongation of the ischemic insult, the impairment of the autophagic flux causes accumulation of p62, an autophagic cargo adapter [21, 35]. Thereafter, p62 can bind to RIPK1 and mediates necrosome assembly and necroptosis [41, 42]. Lethally injured cells tend to undergo apoptosis rather than necroptosis when the function of p62 is suppressed, exemplifying an important role of autophagic modulation in the switching between downstream cell death pathways [41, 42]. Impairment of autophagy accelerates cardiomyocyte loss, adverse ventricular remodeling and heart failure progression following MI [21]. On the other hand, upregulation of autophagy, either genetically or pharmacologically, has been shown to prevent cardiomyocyte death and improve cardiac function [21, 34]. Beneficial effects of apoptosis and/or autophagy modulation in MI models have been reported in many studies [21, 35, 43, 44], whereas the relative impact of MI-induced necroptosis and its therapeutic value are less understood. Consequently, we focus on whether and how necroptosis contributes to the pathophysiology of MI. Moreover, we have addressed the cross-talk between necroptosis and the other forms of cell death. This comprehensive review will be helpful in devising necroptosis-directed therapeutic strategies for MI patients in the near future. Previously, the interventional studies using novel therapeutic strategies to inhibit myocardial cell death have been shown to exert cardioprotective effects in both in vitro and in vivo models [21, 45–51]. Despite the acknowledged benefit conferred by cell death inhibitors on the failing heart, their specific role, in particular necroptosis inhibitors in cases of chronic MI remains unclear. This article comprehensively reviews studies into the context of the necroptosis pathway as well as the interventions inhibiting myocardial cell death from in vitro studies and the preservation of cardiac function from in vivo investigations and clinical reports of chronic MI models. A summary of cell death in experimental models of chronic MI is shown the Table 1.

**Table 1:** Summary of cell death in experimental models of chronic MI

Study model	Methods	Necroptosis	Apoptosis	Autophagy		References
H9c2 cells	OGD for 24 h (16% CO_2_, < 0.1% O_2_)	↑ (3 h) ↓ (after 3 h)	↑ (6 h) ↓ (after 6 h)		↑ (3 h) ↓ (after 3 h)	[29]
H9c2 cells	OGD for 24 h (5% CO_2_, 94% N_2_, 1% O_2_)	↑	N/A		↓	[34]
	OGD for 24 h (5% CO_2_, 94% N_2_, 1% O_2_) + Alliin: 25, 100, 200 μg/ml (pre-treatment)	↓	↓		↑	
H9c2 cells	OGD for 24 h (5% CO_2_, 94% N_2_, 1% O_2_)	↑	N/A		↑ (3 h) ↓ (after 6 h)	[21]
	OGD + RIPK3 overexpression	↑	N/A		N/A	
	OGD + RIPK3 knockdown	↓	N/A		N/A	
	OGD + Beclin1 overexpression	↓	N/A		↑	
	OGD + Beclin1 knockdown	↑	N/A		↓	
NRVCM cells	TNFα + zVAD	↑	N/A		N/A	[33]
MEFs cells	TNFα + Traf2^−/−^	↑	N/A		N/A	[30]
	TNFα + shTraf2	↑	N/A		N/A	
	TNFα + shTraf2 + zVAD	↔	N/A		N/A	
	TNFα + shTraf2 + Nec-1	↓	N/A		N/A	
	TNFα + Ad-Traf2^WT^	↓	N/A		N/A	
	TNFα + Ad-Traf2^ΔR^	↑	N/A		N/A	
	TNFα + Ad-Traf2^ΔR^ + zVAD	↔	N/A		N/A	
	TNFα + Ad-Traf2^ΔR^ + zVAD + Ad-shTRADD	↓	N/A		N/A	
	TNFα + AdTraf2^ΔR^ + Ad-TAK1^ΔN^ + zVAD	↓	N/A		N/A	
	TNFα + AdTraf2^ΔR^ + AdshRIPK3 + zVAD	↓	N/A		N/A	
	TNFα + AdTraf2^ΔR^ + shMLKL + zVAD	↓	N/A		N/A	
	TNFα + Traf2^−/−^ + zVAD	↔	N/A		N/A	
	TNFα + Traf2^−/−^ + Nec-1	↓	N/A		N/A	
	TNFα + Ad-Traf2^ΔR^ + Nec1	↓	N/A		N/A	
Isolated cardiomyocytes	Hypoxic conditions for 72 h (95% N_2_, 5% CO_2_, 0% O_2_)	↑	N/A		N/A	[31]
	Hypoxic conditions + agomiR-325-3p + Z-IETD-FMK	↑	N/A		N/A	
	Hypoxic conditions + antagomiR-325-3p + Z-IETD-FMK	↑	N/A		N/A	
	Hypoxic conditions + siRIPK3 + agomiR + Z-IETD- FMK	↑	N/A		N/A	
	Hypoxic conditions + siRIPK3 + antagomir + Z-IETD-FMK	↔	N/A		N/A	
Cardiac myofibroblasts	sCD74 (0, 0.04, 0.16, 8, 16, 40 nmol/L) + rMIF (8 nmol/L) for 24 h	↑	↔		N/A	[77]
SD rats	Permanent LAD ligation for 4 weeks	↑	↑		↑	[29]
C57BL/6 mice	Permanent LAD ligation for 2 weeks	↑	↑		N/A	[34]
	Permanent LAD ligation for 2 weeks + Alliin: 100 mg/kg IP for 7 days (pre-treatment)	↓	↓		↑	
RIPK3^−/−^ mice	Permanent LAD ligation for 4 weeks	↓	N/A		N/A	[33]
C57BL/6 mice	Permanent LAD ligation for 12 weeks	↑	N/A		↑ (1–3 day) ↓ (after 1 week)	[21]
	Permanent LAD ligation for 12 weeks + RIPK3 knockdown	↓	N/A		N/A	
	Permanent LAD ligation for 12 weeks + RIPK3 overexpression	↑	N/A		N/A	
	Permanent LAD ligation for 12 weeks + Beclin1 knockdown	↑	N/A		↓	
	Permanent LAD ligation for 12 weeks + Beclin1 overexpression	↓	N/A		↑	
C57BL/6 J mice	Permanent LAD ligation	↑	↑		N/A	[31]
	Permanent LAD ligation + Antagomir- 325-3p	↑	↑		N/A	
	Permanent LAD ligation + AgomiR- 325-3p	↓	↓		N/A	
Genetically modified mouse models	Traf2fl/fl-αMHC-Cre	↑	↑		N/A	[30]
	Traf2fl/fl- βMHC-Cre (Traf2 deficient)	↑	N/A		N/A	
	RIPK3^−/−^ + Traf2fl/fl-αMHC-Cre	↓	↑		N/A	
	Permanent LAD ligation for 2 weeks + Traf2fl/ + αMHC-Cre	↑	↑		N/A	
CAD patients	Plasma/serum collected from patient (SCAD, UA, MI)	↑	N/A		N/A	[26]
Patients with HF	Peripheral venous blood samples	↑	N/A		N/A	[38]
Patients with HF	LV samples (CAD)	↑	↔		N/A	[64]
	LV samples (DCM)	↑	↔		N/A	
Patients with HF	LV samples (CAD and DCM)	↑	↑		↑	[63]

CAD, coronary artery disease; DCM, dilated cardiomyopathy; HF, heart failure; IP, intraperitoneal injection; LAD, left anterior descending coronary artery; LV, left ventricle; MEF, mouse embryonic fibroblast; MI, myocardial infarction; MLKL, mixed lineage kinase domain like pseudokinase; N/A, not available; Nec-1, necrostatin 1; NRVCM, neonatal rat ventricular cardiomyocyte; OGD, oxygen–glucose deprivation; RIPK3, receptor-interacting serine/threonine-protein kinase 3; RIPK3^−/−^, receptor-interacting serine/threonine-protein kinase 3 gene knockout; SCAD, stable coronary artery disease; SD, Sprague Dawley; TNFα, tumor necrosis factor alpha; TRADD, tumor necrosis factor receptor type 1-associated DEATH domain protein; Traf2, tumor necrosis factor receptor-associated factor 2; Traft2^−/−^, tumor necrosis factor receptor-associated factor 2 gene knock out; UA, unstable angina; Z-IETD-FMK, caspase 8 inhibitor; zVAD, pan Caspase Inhibitor Z-VAD-FMK; Z-IETD-FM, caspase 8 inhibitor-Z-IE(OMe)TD(Ome)-FMK

## Main text

### Role of programmed necroptosis in the pathogenesis of myocardial infarction

Myocardial ischemia occurs when coronary blood flow to the myocardium is reduced, leading to infarction of the myocardium. The progression of myocardial infarction following myocardial ischemia involves three phases including inflammatory, proliferative and healing phases [9]. The mismatch between oxygen demand and oxygen supply in the ischemic myocardium leads to cellular hypoxia characterized by ATP depletion, metabolic shift from glycolysis to anaerobic respiration and intracellular acidosis. Afterward, ROS production is enhanced, resulting in oxidative injuries which is an important trigger of cell death mechanisms [11, 13, 22, 52]. In addition, previous studies demonstrated the increased levels of TNF-α in the ischemic myocardium [53]. The major source of TNF-α is the mononuclear macrophages residing in the ischemic area [53]. Although TNF-α is not expressed in healthy cardiomyocytes, de novo synthesis of TNF-α by the cardiomyocytes has been demonstrated under ischemic conditions [53]. Persistent upregulation of TNF-α contributes to post-MI cardiac remodeling by promoting cell death and inflammation [33, 34]. It has been shown that TNF-α is a key regulating factor in the inflammatory response during the inflammatory phase. This occurs 1 to 4 days after the myocardium becomes ischemic [54]. TNF-α binds and activates Tumor necrosis factor receptor 1 (TNFR1), leading to the assembly of complex I, which is composed of TNF receptor-associated factor 2 (Traf2), TNFR1-associated death domain protein (TRADD), RIPK1, cellular inhibitor of apoptosis protein-1 (cIAP1), the tumor-suppressor cylindromatosis (CYLD) and the linear ubiquitin chain assembly complex (LUBAC). Binding of TNF-α with TNFR1-related signaling pathways is also involved in the activation of necroptosis-dependent cardiomyocyte death [21]. The inactivation of caspase-8 in complex IIb after the activation of TNFR1 induces the phosphorylation of RIPK1 and RIPK3 to form pro-necrotic complexes or necrosomes. Afterward, the activated p-RIPK3 phosphorylates the mixed lineage kinase domain-like pseudokinase (MLKL). The phosphorylated MLKL is then translocated from the cytoplasm to the plasma membrane, resulting in MLKL-mediated membrane permeation and cardiomyocyte death [21]. In addition to the canonical RIPK1-RIPK3-MLKL pathway, Zhang and colleagues reported that RIPK3 also mediated myocardial necroptosis through the activation of Ca^2+^/calmodulin-dependent protein kinase (CaMKII) in cardiac I/R model [89]. It has been reported that cardiomyocyte death induced by necroptosis also triggers a greater inflammatory response followed by accumulation of granulocytes and mononuclear phagocytes in the infarct tissue several days after MI [9].

The proliferative phase begins a couple of days later followed by an inflammatory phase and lasts for 3 to 4 weeks after MI [9]. During this phase, transforming growth factor-β (TGF-β) is produced by cardiomyocytes or macrophages in the infarcted myocardium [55]. TGF-β exerts anti-inflammatory actions, myofibroblast activation and differentiation as well as the regulation of angiogenesis and vascular maturation, resulting in the remodeling of the infarcted heart [55]. Finally, the healing phase begins 2 to 6 weeks of MI progression [9]. In the healing phase, the infarcted myocardium undergoes scar maturation, the scar being composed of a dense collagen-based extracellular matrix, myofibroblasts, cardiac stem cells and neovascularized vessels [55]. It has been demonstrated that cardiomyocyte necroptosis is involved in inflammation, oxidative stress-related myocardial injury, cardiac fibrosis and cardiomyocyte proliferation after MI [56]. Insights regarding the mechanistic link between necroptosis and pathophysiological sequelae of MI (i.e. the inflammatory process and the healing process) are emerging. Myocardial ischemia induces death of cardiomyocytes and triggers an inflammatory response [10, 20, 22], resulting in progressive inflammatory cell infiltration and fibrosis [9]. The loss of functional cardiomyocytes and increased fibrosis are responsible for cardiac contractile dysfunction and failure [24–27]. Of note, necroptosis is inherently immunogenic since it leads to the disruption of plasma membrane, thereby causing the release of pro-inflammatory biomolecules (collectively called damage-associated molecular patterns, DAMPs) [13, 57, 58]. In addition, ongoing progression of chronic MI has been associated with diverse types of cell death mechanisms [13].

It has been shown that several types of cell death mechanism are primarily involved in MI progression [13]. Both apoptosis and necrosis have already been identified as being involved [59]. Apoptosis is a highly regulated cell death mechanism [13, 52], which significantly contributes to cardiomyocyte death during the acute onset of MI progression and predominantly originates in the peri-infarction area [21, 29, 34, 52]. In contrast, necrosis has been described as passive unregulated cell death [13, 24]. Recent studies have demonstrated that necroptosis is a regulated form of necrosis which is mediated by death receptor signaling [57]. During ischemia, various types of death receptors such as TNFR1, Fas ligand receptor (FasR), TNF-related apoptosis-inducing ligand receptor (TRAIL-R) or toll like receptors (TLRs) are stimulated in the ischemic myocardium and the surrounding tissues [13, 59–61]. As reported by Linkermann et al., TNF-α is a key regulator of inflammation and repair in cardiac tissue [61]. Although TNF-α/TNFR1 signaling is a potent trigger of necroptosis, other cytokines in the TNF family can also initiate necroptosis through various pattern recognition receptors (PRRs) [13, 61], with Toll-like receptors (TLRs) being one of the most intensively studied subgroup. The study of Yang et al. demonstrated that TLR4 was responsible for myocardial inflammation in myocarditis, cardiac I/R and MI [62]. The activated TNF/TLR signaling, in turn, recruited RIPK1 to compose a necroptosis-inducing protein complex in a manner similar to the better characterized TNF-α/TNFR1-mediated pathway [54, 56, 57]. Cumulative evidence suggested that necroptosis is closely associated with pathologies of MI progression [13, 26, 38, 63, 64]. RIPK1-RIPK3-MLKL dependent necroptosis was found to be regulated in cardiomyocytes which could be enhanced by the disruption of apoptosis and/or impairment of autophagy flux [21, 29, 65]. As a result, necroptosis is induced under chronic ischemic conditions, leading to impaired cardiac contractility and functioning with increased risk of cardiovascular mortality.

**Fig. 2:**
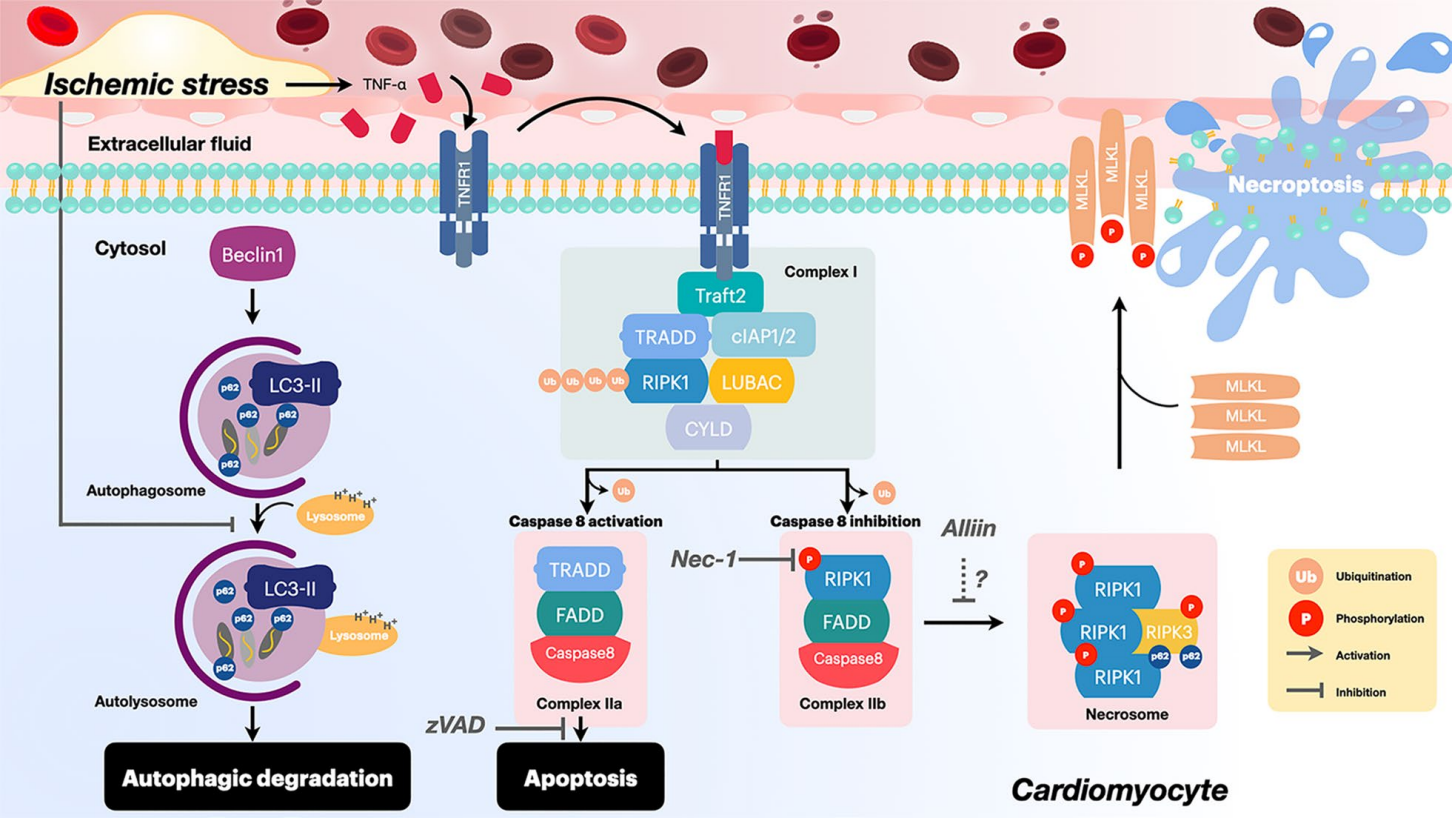
Regulatory mechanism of necroptosis. Under ischemic conditions, TNF-α activated TNFR1 then triggered the assembly of complex I. The activation of caspase-8 would result in cardiomyocyte loss through apoptosis pathway while, the inactivation of caspase-8 in complex Iib induced the phosphorylation of RIPK1 and RIPK3 and formed pro-necrotic complexes or necrosomes. Then, the activated p-RIPK3 would phosphorylate MLKL to p-MLKL which will be translocated from cytoplasm to the plasma membrane and mediate membrane breakdown, leading to necroptotic cell death. Under prolongation of ischemic insult, the impairment of the autophagic machinery leads to accumulation of p62 which causes necroptosis dependent cell death. In addition, the intervention with several cell death modulators could improve cardiomyocyte viability under ischemic conditions. Pan-Caspase inhibitor Z-VAD acts as an effective caspase inhibitor resulting in prevention of apoptosis. Necroptosis is inhibited by Nec-1 which inhibits the activity of RIPK1 while Alliin prevents necroptosis cell death by mitigating necroptosis markers. TNF-α: tumor necrosis factor-α; TNFR1: tumor necrosis factor receptor 1; RIPK1: receptor-interacting serine/threonine-protein kinase 1; RIPK3: receptor-interacting serine/threonine-protein kinase 3; MLKL: mixed lineage kinase domain-like; p-MLKL: phosphorylated-mixed lineage kinase domain-like; Traf2: tumor necrosis factor receptor associated factor 2; cIAP1/2: cellular inhibitors of apoptosis 1 and 2; LUBAC: linear ubiquitin chain assembly complex; CYLD: the tumor-suppressor cylindromatosis; TRADD: TNFR1-associated death domain protein; FADD: fas-associated protein with death domain; Nec-1: necrostatin 1; zVAD: pan-Caspase inhibitor carbobenzoxy-valyl-alanyl-aspartyl-[O-methyl]-fluoromethylketone; LC3-II: lipid modified form of microtubule-associated protein 1A/1B-light chain 3; p62: ubiquitin-binding protein p62

Maladaptive inflammation is a key driver of post-MI remodeling and deterioration of cardiac performance [54]. Both electrical and mechanical defects evolve in the inflamed myocardium as a consequence of progressive cardiomyocyte loss and abnormal extracellular matrix deposition [66, 67]. Necroptosis is known as one of the regulated forms of necrosis that initiate a robust inflammatory response by a release of DAMPs from the disruption of the plasma membrane [58]. The consequence following RIPK3-mediated necroptosis is the aggravation of inflammatory processes in various organs, including the heart, intestine and skin [58, 68–70]. In the heart tissue of MI mice, the boundary between the infarcted area and the non-infarcted area had significantly increased inflammatory cells infiltration [25]. Several studies demonstrated that necroptosis could triggers neutrophils, macrophages, T-cells and the other inflammatory cell infiltration and also aggravates an immune response which coordinates with the process of tissue repair [13, 50, 57, 71, 72]. In contrast to necroptosis, apoptosis causes no disruption of the plasma membrane, and only membrane blebbing (zeiosis) occurs [11–13]. As a result, the secretion of inflammatory cytokines by apoptotic cells is very limited [13, 57, 73]. Therefore, apoptosis could trigger macrophage clearance, but less likely to further induce inflammation. In contrast, since the plasma membrane is ruptured, necroptosis could trigger various types of immune cells, leading to more detrimental inflammation. Therefore, inhibition of necroptosis may be a novel therapeutic target for the treatment of the necroptosis-activated pathophysiology underlying MI. A schematic diagram summarizing the regulatory mechanism of necroptosis is shown in Fig. 2.

### Targeting necroptosis in the chronic MI model

Necroptosis has been reported to be involved in the death of cardiomyocytes during the period of myocardial ischemia as well as post-MI remodeling [74–77]. Interestingly, necroptosis also occurred in other cardiac resident cells including the coronary endothelial cells and cardiac myofibroblasts [74–77]. Apart from the cardiovascular system, necroptosis involves in the pathophysiological processes in other organs, particularly following ischemic and/or inflammatory insults [57, 68, 72, 73, 75, 78]. Specifically, necroptosis has been demonstrated in a wide range of non-cardiac cell types such as pulmonary epithelial cells, intestinal epithelial cells, renal tubular epithelial cells and neuronal cells [57, 71–73, 78].

Apoptosis and necroptosis pathways share some signaling molecules downstream to the TNFR activation by TNF-α [13, 50, 73]. It has been shown that TNF-α is a key trigger of apoptosis and necroptosis under inflammatory and stress-related conditions [73]. However, the precise mechanism determining whether the cells would die from apoptosis or necroptosis remains an issue under intense investigation [71]. Growing evidence emphasizes the regulation of caspase-8 activities as a pivotal role in necroptosis regulation [13, 50, 71, 73]. The FADD/caspase-8 signaling pathway negatively regulates necroptosis by preventing the formation of RIPK1-RIPK3 heterodimer in complex IIb, thereby preventing the induction of RIPK3-dependent necroptosis [79]. The activation of caspase-8 would result in cardiomyocyte loss through the apoptosis pathway, whereas the inhibition of caspase-8 in complex IIb induced the phosphorylation of RIPK1 and RIPK3 and formed pro-necrotic complexes or necrosomes, leading to necroptosis [13, 50, 79]. Accordingly, the regulation of caspase-8 activities is essential for determining the fate of cell death between apoptosis and necroptosis [13, 58]. However, the precise mechanisms or mediators which ultimately determine the dominant cell death pathway remains unclear. The comprehensively summarized key features used for differentiation between apoptosis and necroptosis in the experimental setting is shown the Table 2.

**Table 2:** Markers used for differentiation between apoptosis and necroptosis in the experimental settings

	Apoptosis	Necroptosis	References
Morphology	Cytoplasmic shrinkage Chromatin condensation (pyknosis) Nuclear fragmentation (karyorrhexis) Plasma membrane blebbing (zeiosis) Shedding of apoptotic bodies	Increasingly translucent cytoplasm Swelling of organelles Membrane permeabilization Increased cell volume (oncosis) Mild chromatin condensation (nuclei remain intact)	[11, 13, 20, 71, 73, 87]
Death execution events	Caspase-3 execution pathway causes cell shrinkage, chromosomal condensation and DNA fragmentation	MLKL phosphorylation and translocation to the plasma membrane causes membrane permeabilization	[11–13, 20, 27, 71, 73, 87, 88]
Death regulatory factors	BID, BAX, Bcl-2, Cytochrome c APAF1, FADD, Caspase-8, Caspase-9	RIPK1, RIPK3	[11–13, 20, 27, 58, 71, 73, 87, 88]
Death execution factors	Caspase-3 Caspase-7	p-MLKL	[11–13, 20, 27, 58, 71, 73, 87, 88]
Methods for evaluation in cardiomyocytes	TUNEL assays DNA laddering Annexin V positive Caspase-3/7 activity assay	PI staining (staining with impermeant dyes) HMGB1 release LDH assay Detection of RIPK1, RIPK3 and MLKL	[71, 87]

APAF1, Apoptotic protease activating factor 1; Bax, BCL2 associated X protein; BCL-2, B-cell lymphoma 2 protein; BID, BH3-interacting domain death agonist; FADD, Fas-associated protein with death domain; HMGB1, high mobility group box 1; LDH, Lactate dehydrogenase; MLKL, Mixed lineage kinase domain like pseudokinase; p-MLKL, the phosphorylated form of mixed lineage kinase domain like pseudokinase; RIPK1, receptor-interacting serine/threonine-protein kinase 1; RIPK3, receptor-interacting serine/threonine-protein kinase 3; TUNEL, terminal deoxynucleotidyl transferase dUTP nick end labeling

Reports from in vitro, in vivo and clinical studies supported the concept that necroptosis is associated with cardiomyocyte loss, adverse cardiac remodeling, impaired left ventricular function and heart failure [21, 29–31]. However, there are only a few investigations into the role of necroptosis in chronic MI models. In this review, the beneficial effects of genetic modification and pharmacological intervention targeting necroptosis signaling pathways and their roles in cardiac function of MI models from various studies including in vitro and in vivo studies are comprehensively summarized and discussed.

### Role of necroptosis in MI mimicking conditions: reports from in vitro studies

MI-induced myocardial cell death could result from significant and sustained ischemia due to inadequate coronary perfusion [3]. Various forms of cell death including necroptosis, necrosis and apoptosis have been suggested as determinants in the fate of cardiomyocytes after MI [65]. To mimic the MI conditions in in vitro studies, cardiomyocytes were subjected to conditions of oxygen and glucose deprivation (OGD). Under OGD conditions, necroptosis was obviously activated in cardiomyocytes as evidenced by increasing necroptosis markers including RIPK1, RIPK3 and MLKL [21, 29, 31, 34]. RIPK1-RIPK3-MLKL interaction subsequently disrupted plasma membrane integrity as the key step in necroptosis execution. Loss of cardiomyocytes due to necroptosis was represented by the increase in number of PI-positive cardiomyocytes in a time-dependent manner [21].

To investigate whether necroptosis could be triggered by the stimulation of death receptors, TNFR1 signaling mediated by TNF-α has been widely studied [30, 33]. Under conditions of MI injury, a considerable amount of inflammatory response was found to occur in the myocardium and surrounding tissues [54]. In general, MI had contributed to myocardium ischemia and anoxia, followed by the activation of cardiomyocytes and myocardial local mononuclear macrophages and production of large numbers of TNF-α [54]. Recent studies demonstrated that TNF-α mediated cardiomyocyte necroptosis occurred via the activation of RIPK1, RIPK3 and MLKL cascades [30, 33].

Traf2 was also identified as playing a vital role in cardiomyocyte survival and homeostasis through the regulation of necroptosis and apoptosis [30]. Genetic deletion of Traf2 in mice led to premature mortality as a consequence of severe developmental immunodeficiency, and increased cellular sensitivity to TNF-α dependent cell death [30]. Genetic modification of Traf2 deficient mice resulted in aggregated necroptosis signaling through RIPK1-RIPK3-MLKL interaction [30], whereas overexpression of Traf2 could improve cardiomyocyte viability in TNF-α induced necroptosis [30]. In the view of these admittedly limited in vitro reports, necroptosis was clearly present in MI mimicking conditions which were induced by the OGD condition or the modulation of death receptor signaling. All of these reports are summarized in Additional file 1: Table S1.

### Modulating necroptosis as a potential therapeutic target in MI: reports from in vitro studies

Recently, it has been demonstrated that the small molecules called necrostatins could significantly inhibit the kinase activity of RIPK1, which potentially suppress necroptosis process [47, 80–83]. Necrostatin-1 (Nec-1), which belongs to the necrostatin group, is identified as a potential inhibitor of RIPK1 [84]. Nec-1 has a specific allosteric inhibitory effect on the hydrophobic pocket of the RIPK1 kinase domain [84, 85]. Nec-1 not only inhibits the recruitment of RIPK1 to FADD in complex II, but also prevent RIPK1-RIPK3 interaction [58]. Thus, Nec-1 effectively inhibited necroptosis via suppressing the RIPK1-RIPK3-MLKL cascade in cardiomyocytes [47, 58, 81]. Interestingly, Wang et al. (2012) demonstrated that Nec-1 could also suppress autophagy and apoptosis in mice with traumatic brain injury models [82]. Moreover, Liu et al. also found that Nec-1 suppressed apoptotic death in the skin flap I/R model [81]. These findings suggest the interplay between Nec-1 and multiple cell death pathways in the non-cardiomyocyte models. Nevertheless, whether and to what extent necroptosis inhibitors affect other types of cell death in cardiomyocytes, particularly in MI-related model, remains unknown.

The reports on interventions to attenuate the loss of cardiomyocytes and cardiac remodeling regarding necroptosis in MI mimicking conditions from in vitro studies are shown in Additional file 1: Table S1. It has been shown that Nec-1 an inhibitor of RIPK1 activity, is a potential inhibitor of necroptosis [86]. Nec-1 has the potential to provide a protective effect against TNF-α mediated necroptosis, while the intervention using pan-Caspase inhibitor Z-VAD-FMK could not [30]. In addition, Nec-1 also improved cell viability in Traf2-deficient MEF cells which had undergone TNF-α induced necroptosis [30]. As shown earlier, Traf2 is a key intracellular signaling mediator that acts downstream of not only TNF-α, but also various members of the TNF-α superfamily [30]. Therefore, the elimination of TNF-α signaling leads to increased cell viability of Traf2-deficient MEF cells [30]. All of these findings suggested that suppression of the necroptotic signaling via Nec-1 could serve as a new therapeutic target for prevention of the loss of cardiomyocytes under MI mimicking conditions.

In addition to Nec-1 treatment, the organic compound *S*-allyl-cysteine sulfoxide (alliin) was shown to promote cell survival in H9c2 cells in the OGD induced necroptosis model by decreasing RIPK1, and RIPK3, increasing autophagy and decreasing apoptosis in a dose-dependent manner [34]. Notwithstanding, there remains a lack of investigations into the type of cell death pathways which play the most dominant role in the MI associated model. Thus, further study is needed to investigate the role and proportion of each cell death pathway in the MI model.

### Role of necroptosis in MI conditions: reports from in vivo studies

In the same way as necroptosis was detected in OGD conditions in in vitro studies, necroptosis was also shown to exist in in vivo studies (Additional file 1: Table S2). Permanent left anterior descending coronary artery (LAD) ligation has been used for the experimental chronic MI model in rats. In addition to cardiac pathologies including cardiac fibrosis, adverse cardiac remodeling and cardiac dysfunction were observed in chronic MI [21, 29, 31, 33, 34], necroptosis was also shown to persistently occur a week after the progression of MI [21]. Necroptosis markers including RIPK1 and RIPK3 were increased after a week of LAD ligation when compared with sham rats [21]. These findings supported that necroptosis was timely activated in response to ischemic conditions, and it also instigated the loss of cardiomyocytes**,** resulting in cardiac dysfunction after MI.

Another element involved in necroptosis is Traf2 which plays a pivotal role in cardiomyocyte homeostasis by modulating necroptosis and apoptosis [30]. Traf2 also mediates the nuclear factor-κB (NFκB) independent survival pathway in the heart via the suppression of RIPK1-RIPK3-MLKL necroptotic signaling [30]. Genetic modification of Traf2 deficient mice led to cardiomyocyte death, pathological cardiac remodeling, and cardiac dysfunction [30]. These findings suggested that Traf2 plays a beneficial role in myocardial survival and homeostasis by suppressing necroptosis signaling. In addition, the evidence from studies into necroptosis and MI pathologies indicated that the inhibition or ablation of necroptotic markers including RIPK3 provided beneficial effects in RIPK3 knockout (RIPK3^−/−^) mice as well as RIPK3 knockdown (RIPK3^−/+^) mice via the amelioration of the MI-induced cardiac remodeling and dysfunction as well as in heart failure [21, 33]. All of these findings suggested that inhibition of necroptosis signaling might have a potential therapeutic role in MI patients. All of these reports are summarized in Additional file 1: Table S2.

### Modulating necroptosis as a potential therapeutic target in MI: reports from in vivo studies

Consistent with the findings from an in vitro model, alliin also improved LV systolic function and increased animal survival rate via the amelioration of both necroptosis cell death and the TUNEL positive cells caused by MI in an in vivo model (Additional file 1: Table S2) [34]. Alliin exerts cardioprotective effects not only by inhibiting both necroptosis and apoptosis, but also by escalating autophagy machinery in an in vitro hypoxic model and an in vivo MI model [34].

### Role of necroptosis in MI conditions: reports from clinical studies

Limited clinical studies are available regarding the roles of necroptosis in MI patients. In HF patients, it has been demonstrated that various necroptosis markers including RIPK1, RIPK3 and phosphor-MLKL were upregulated in these patients [26, 38, 63, 64]. These findings suggested that necroptosis could be extensively detected in both blood samples [26, 38] and ventricular tissue samples [63, 64] obtained from patients with HF resulting from coronary artery disease (CAD). RIPK3, a key determinant of necroptosis, was shown to be markedly increased and showed a positive correlation with the severity of HF [38], while it was found to have a low level of expression in the non-HF samples [26, 38, 63, 64]. In one cross-sectional study, the plasma levels of the necroptotic marker RIPK3 were correlated with the severity of coronary artery disease (i.e. low in stable coronary disease, higher in unstable angina, and highest in MI) [26]. Moreover, an association between certain genetic variants of RIPK3 and higher mortality in HF patients was reported [38]. Regardless of this clue of clinical significance, there is still no direct prognostic study which prospectively investigates the correlation between the extent of necroptosis and the clinical outcomes in MI/HF patients. A summary of these clinical reports is presented in Additional file 1: Table S3.

## Conclusions

There is growing evidence to demonstrate that sustained inadequate coronary perfusion during MI progression leads to cardiomyocyte loss caused by necroptosis. Loss of cardiomyocytes after MI is critical in the subsequent adverse remodeling, cardiac dysfunction and heart failure. The necroptosis markers including RIPK1 and RIPK3 can be extensively detected in the blood and the heart in both experimental models and in patients with MI. Necroptosis markers also show a correlation with the severity of the disease which could imply that necroptosis is related to the pathology of MI progression. At this time, several pharmacological interventions have been shown to preserve the loss of cardiomyocytes, cardiac remodeling and dysfunction caused by necroptosis in MI. These limited findings support necroptosis modulation by necroptosis inhibition as targets to enhance the cell viability and cardiac function in MI models. Due to limited reports available, further studies are required to clarify the pathophysiological roles of necroptosis and to warrant the potential use of necroptosis modulators as therapeutic targets in MI.

## Supplementary Information

**Additional file 1.** Supplementary tables (Tables S1–S3) comprehensively summarizing in-depth information of findings from the key studies included in the review.

## Data Availability

Not applicable.

## Abbreviations

CAD: Coronary artery disease
cIAP1: Cellular inhibitor of apoptosis protein-1
CVDs: Cardiovascular diseases
CYLD: The tumor-suppressor cylindromatosis
FasR: Fas ligand receptor
HF: Heart failure
LAD: Left anterior descending coronary artery
LUBAC: Linear ubiquitin chain assembly complex
MLKL: Mixed lineage kinase domain like pseudokinase
MI: Myocardial infarction
Nec-1: Necrostatin-1
NFκB: Nuclear factor-κB
OGD: Oxygen and glucose deprivation
RIPK1: Receptor-interacting serine/threonine-protein kinase 1
RIPK3: Receptor-interacting serine/threonine-protein kinase 3
RIPK3^−^^/^^−^: Receptor-interacting serine/threonine-protein kinase 3 knockout
RIPK3^−^^/^^+^: Receptor-interacting serine/threonine-protein kinase 3 knockdown
TGF-β: Transforming growth factor beta
TLRs: Toll like receptors
TNF-α: The tumor necrosis factor alpha
TNFR1: Tumor necrosis factor receptor 1
TRADD: Tumor necrosis factor receptor type 1-associated DEATH domain protein
Traf2: Tumor necrosis factor receptor-associated factor 2
TRAIL-R: TNF-related apoptosis-inducing ligand receptor
