# Supplementary material for: Targeting necroptosis as therapeutic potential in chronic myocardial infarction
Source: J Biomed Sci. 2021 Apr 9;28:25. doi: 10.1186/s12929-021-00722-w (PMC8034148; doi:10.1186/s12929-021-00722-w)
Supplement: Supplementary file 1 — Additional file 1. Supplementary tables (Tables S1–S3) comprehensively summarizing in-depth information of findings from the key studies included in the review. [file 12929_2021_722_MOESM1_ESM.docx]

**Supplementary Table 1.** Necroptosis in MI mimicking conditions: reports from *in vitro* studies.

| **Study model** | **Methods** | **Major finding** | | | | **Interpretation** | **Ref.** |
| --- | --- | --- | --- | --- | --- | --- | --- |
|  |  | **Cell viability (%)** | **Necroptosis** | **Apoptosis** | **Other biochemical markers** |  |  |
| H9c2 cells | OGD for 24 h  (16% CO_2_, <0.1% O_2_) | ↓ | ● ↑ PI-positive cells  ● ↑ RIPK1, RIPK3   (3 h)  ● ↓ RIPK1, RIPK3  (after 3 h) | ● ↑ Apoptotic cells  ● ↑ CCP3/CP3 (6 h)  ● ↓ CCP3/CP3  (after 6 h) | ● ↑ Autophagosomes  (3 h)  ● ↑ LC3 II (3 h)  ● ↓ LC3 II (after 3 h)  ● ↑ LC3 II (after 15 h)  ● ↑ Aggresome  ● ↑ p62 | OGD mediated necroptosis via increased RIPK1, RIPK3 and also induced autophagy and apoptosis. | (29) |
| H9c2 cells | OGD for 24 h  (5% CO_2_, 94% N_2_, 1% O_2_) | ↓ | ● ↑ PI-positive cells  ● ↑ RIPK1, RIPK3  ● ↑ Traf2  ● ↑ CCP3 | N/A | ● ↓ Beclin1  ● ↓ ATGs proteins  ● ↓ PPARγ | OGD mediated necroptosis via increasing RIPK1, RIPK3, Traf2 and downregulating autophagy. | (34) |
| H9c2 cells | OGD for 24 h  (5% CO_2_, 94% N_2_, 1% O_2_) | ↓ | ● ↑ PI-positive cells  ● ↑ RIPK1, RIPK3  ● ↑ p-MLKL | N/A | ● ↑ Beclin1  ● ↑ LC3 II/I  ● ↓ p62 (3 h)  ● ↑ p62 (6 h)  ● ↑ Autophagosomes  ● ↑ Autolysosomes (3 h)  ● ↓ Autolysosomes   (12 h) | ● OGD mediated necroptosis via increased RIPK1, RIPK3 and p-MLKL signaling cascade and impaired autophagy flux.  ● Impaired autophagy flux enhanced necroptosis in OGD injuries through decreased Beclin1 expression. | (21) |
|  | **Pre-treatment with:**  ● RIPK3 overexpression | ↓ | ● ↑ PI-positive cells | N/A | N/A |  |  |
|  | ● RIPK3 knockdown | ↑ | ● ↓ PI-positive cells | N/A | N/A |  |  |
|  | ● Beclin1 overexpression | ↑ | ● ↓ RIPK3  ● ↓ PI-positive cells | N/A | ● ↑ LC3 II/I  ● ↑ Autophagosome  ● ↑ Autophagolysosome |  |  |
|  | ● Beclin1 knockdown | ↓ | ● ↑ RIPK3  ● ↑ PI-positive cells | N/A | ● ↓ LC3 II/I  ● ↓ Autophagosome  ● ↓ Autophagolysosome |  |  |
| Neonatal rat ventricular cardiomyocytes (NRVCMs) | TNFα + zVAD-fmk  **Pre-treatment with:**  ● AdV RIPK3 + TNFα + zVAD-fmk (50 μM and 100 ng/mL respectively for 4 hr) | ↓ | ● ↑ PI-positive cells  ● ↑ p-RIPK3  ● ↑ RIPK3 ubiquitination  ● ↑ RIPK1/RIPK3 complexes  ● ↓ RIPK1 | N/A | N/A | RIPK3 overexpression promoted TNFα mediated necroptosis via increased RIPK1/RIPK3 complex and decreased RIPK1 level. | (33) |
| Mouse embryonic fibroblasts (MEFs) | TNFα stimulation  (10 ng/ml for 4h)  **Deletion of Traf2**  ● Traf2^-/-^ | ↓ | ● ↑ PI-positive cells  ● ↑ HMGB1  ● ↑ PARP  ● ↑ CP8 | N/A | N/A | ● TNFα mediated necroptosis via the activation of RIPK1 and MLKL in Traf2 knockout cells.  ● Traf2 overexpression suppressed TNFα mediated necroptosis via the downregulation of RIPK1, RIPK3, and MLKL.  ● Overexpression of Traf2 lacking the RING E3 ubiquitin ligase domain promoted TNFα-induced necroptosis via RIPK3 and MLKL signaling pathway.  ● TRADD and TAK1 play an important role in TNFα mediated necroptosis. | (30) |
|  | ● shTraf2 | ↓ | ● ↔ PI-positive cells  ● ↑ HMGB1  ● ↑ PARP  ● ↑ CP8 | N/A | N/A |  |  |
|  | ● shTraf2 + zVAD | ↔ | ● ↑ PI-positive cells  ● ↑ HMGB1  ● ↓ PARP  ● ↑ CP8 | N/A | N/A |  |  |
|  | ● shTraf2 + Nec-1 | ↑ | ● ↓ PI-positive cells  ● ↓ HMGB1  ● ↓ PARP  ● ↑ CP8 | N/A | N/A |  |  |
|  | **Overexpression of Traf2**  ● Ad-Traf2^WT^ | ↑ | ● ↓ PI-positive cells  ● ↓ HMGB1 | N/A | N/A |  |  |
|  | **Overexpression of Traf2 lacking the RING E3 ubiquitin ligase domain**  ● Ad-Traf2^ΔR^ | ↓ | ● ↑ PI-positive cells  ● ↑ HMGB1  ● ↑ RIPK1, RIPK3  ● ↑ FADD | N/A | N/A |  |  |
|  | ● Ad-Traf2^ΔR^ + zVAD | ↔ | ● ↔ PI-positive cells  ● ↑ HMGB1 | N/A | N/A |  |  |
|  | **Deletion of TRADD**  ● Ad-Traf2^ΔR^ + zVAD + Ad-shTRADD | ↑ | ● ↓ PI-positive cells  ● ↓ HMGB1  ● ↓ Necrosome formation | N/A | N/A |  |  |
|  | **Overexpression of TAK1**  ● AdTraf2^ΔR^ + Ad-TAK1^ΔN^ + zVAD | ↑ | ● ↓ PI-positive cells  ● ↓ HMGB1  ● ↓ Necrosome formation | N/A | N/A |  |  |
|  | **Deletion of RIPK3**  ● AdTraf2^ΔR^ + AdshRIPK3 + zVAD | ↑ | ● ↓ PI-positive cells  ● ↓ HMGB1 | N/A | N/A |  |  |
|  | **Deletion of MLKL**  ● AdTraf2^ΔR^ + Lv-shMLKL + zVAD | ↑ | ● ↓ PI-positive cells  ● ↓ HMGB1 | N/A | N/A |  |  |
| Cardiomyocytes isolated from adult male C57BL/6J mice | Hypoxic conditions for 72 h  (95% N_2_, 5% CO_2_, 0% O_2_) | ↓ | ● ↑ RIPK1, RIPK3  ● ↑ p-MLKL | N/A | N/A | OGD mediated necroptosis via the upregulation of RIPK1, RIPK3, p‐MLKL and downregulation of miR‐325‐3p. | (31) |
|  | **Pre-treatment with:**  ● AgomiR-325-3p +  Z-IETD-FMK | ↑ | ● ↑ RIPK1, RIPK3  ● ↑ p-MLKL | N/A | N/A |  |  |
|  | ● AntagomiR- 325-  3p + Z-IETD-FMK | ↓ | ● ↑ RIPK1, RIPK3  ● ↑ p-MLKL | N/A | N/A |  |  |
|  | ● siRIPK3 + agomiR +  Z-IETD- FMK | ↑ | ● ↓ RIPK1, RIPK3  ● ↓ p-MLKL | N/A | N/A |  |  |
|  | ● siRIPK3 + antagomiR +  Z-IETD-FMK | ↓ | ● ↔ RIPK1, RIPK3  ● ↔ p-MLKL | N/A | N/A |  |  |
| Cardiac myofibroblasts | **Recombinant MIF and sCD74 induced cell death**  ● sCD74  (0, 0.04, 0.16, 8, 16, 40 nmol/L) + rMIF (8 nmol/L) for 24hr. | ↓ | ● ↑ RIPK3 | ● ↔ CCP/CP3  ● ↓ p-AKT | ● ↑ Fibronectin-1 genes  ● ↑ NF-κB related gene  ● ↑ pp38 | Recombinant MIF and sCD74 mediated necroptosis via increased RIPK3 which could be prevented by necrostatin-1s. | (77) |
|  | ● sCD74 + rMIF + Nec-1s for 24hr. | ↑ | N/A |  | N/A |  |  |
| H9c2 cells | OGD for 24 h  (5% CO_2_, 94% N_2_, 1% O_2_)  **Pre-treatment with**:  ● Alliin:  25, 100, 200 μg/ml  for 4h | ↑ | ● ↓ PI-positive cells (dose dependent)  ● ↓ RIPK1, RIPK3  ● ↓ Traf2 | ● ↓ CCP3 | ● ↑ Beclin1  ● ↑ LC3II  ● ↑ ATG4A, ATG4c, ATG4d, ATG9A, ATG7 and ATG16L2  ● ↑ p62  ● ↑ PPARγ | ● Pre-treatment with Alliin inhibited OGD induced necroptosis through decreasing RIPK1, RIPK3 and Traf2 in a dose-dependent manner.  ● Alliin promoted autophagy, inhibited OGD induced apoptosis in a dose-dependent manner. | (34) |
| Mouse embryonic fibroblasts (MEFs) | TNFα stimulation  (10 ng/ml for 4h)  **Pre-treatment with**:  ● Traf2^-/-^ + zVAD | ↔ | ● ↔ PI- positive cells  ● ↑ HMGB1  ● ↑ CP8  ● ↓ PARP | N/A | N/A | ● Nec-1 alleviated TNFα mediated necroptosis in Traf2 deficit and Traf2 lacking the RING E3 ubiquitin ligase domain cells.  ● zVAD did not alleviate TNFα mediated necroptosis in Traf2 deficit cells. | (30) |
|  | ● Traf2^-/-^ + Nec-1 | ↑ | ● ↓ PI-positive cells  ● ↓ HMGB1  ● ↓ PARP  ● ↑ CP8 | N/A | N/A |  |  |
|  | ● Ad-Traf2^ΔR^  + Nec1 | ↑ | ● ↓ PI-positive cells  ● ↓ HMGB1  ● ↓ PARP  ● ↑ CP8 | N/A | N/A |  |  |

ATGs, the Autophagy-related genes; CCP3, cleaved caspase 3; CD74, cluster of differentiation 74; CP3, caspase 3; CP8, caspase 8; FADD, fas-associated protein with death domain; HMGB1, high mobility group box 1; LC3 I, the cytosolic form of microtubule-associated protein 1A/1B-light chain 3; LC3 II, the lipid modified form of microtubule-associated protein 1A/1B-light chain 3; miR‐325‐3p, microRNA-325-3p; MIF, macrophage migration inhibitory factor; MLKL, mixed lineage kinase domain like pseudokinase; Nec-1, necrostatin 1; Nec-1s, necrostatin-1 stable; N/A, not available; OGD, oxygen-glucose deprivation; p-AKT, phosphorylated Akt; p-MLKL, the phosphorylated form of mixed lineage kinase domain like pseudokinase; p-RIP3, the phosphorylated form of receptor-interacting serine/threonine-protein kinase 3; p62, sequestosome 1; PARP, poly (ADP-ribose) polymerase; PI, propidium iodide; PPARγ, peroxisome proliferator-activated receptor gamma; RIPK1, receptor-interacting serine/threonine-protein kinase 1; RIPK3, receptor-interacting serine/threonine-protein kinase 3; TAK1, transforming growth factor-β-activated kinase 1; TNFα, tumor necrosis factor alpha; TRADD, tumor necrosis factor receptor type 1-associated DEATH domain protein; Traf2, tumor necrosis factor receptor-associated factor 2; Traft2^-/-^, tumor necrosis factor receptor-associated factor 2 gene knock out; zVAD, pan Caspase Inhibitor Z-VAD-FMK; Z-IETD-FM, caspase 8 inhibitor-Z-IE(OMe)TD(OMe)-FMK

**Supplementary Table 2.** Necroptosis in chronic MI: reports from *in vivo* studies.

| **Study model** | **Methods** | **Major finding** | | | | **Interpretation** | **Ref.** |
| --- | --- | --- | --- | --- | --- | --- | --- |
|  |  | **Cardiac morphology / Function** | **Necroptosis** | **Apoptosis** | **Other biochemical markers** |  |  |
| Sprague-Dawley rats (male, 180-220 g) | Permanent LAD ligation for 4 weeks | ● ↑ MI size  ● ↓ LVFS, LVEF | **At 1 week**  ● ↑ PI positive cells (MI>peri MI)  **At 4 weeks**  ● ↑ PI positive cells (MI>peri MI) | ● ↑ TUNEL positive cells  (peri-MI>MI)  ● ↑ TUNEL positive cells (MI>peri-MI) | ● ↑ LC3 II (MI>peri-MI)  ● ↑ LC3 II (peri-MI>MI | Chronic MI mediated necroptosis, apoptosis and autophagy leading to LV dysfunction. | (29) |
| C57BL/6 mice  (male, 8-10 wk, 18-20 g) | Permanent LAD ligation for 2 weeks | ● ↓ Survival rate (~50%)  ● ↓ LVFS, LVEF | ● ↑ PI positive cells  ● ↑ RIPK1, RIPK3  ● ↑ Traf2 | ● ↑ TUNEL positive cells  ● ↑ CCP3 | ● ↓ Cells viability | Chronic MI mediated necroptosis via increased RIPK1, RIPK3 and Traf2 as well as apoptosis leading to LV dysfunction and increased mortality. | (34) |
| Genetically modified mouse models  (RIPK3^-/-^ mice) | Permanent LAD ligation for 4 weeks  **Pre-treatment with:**  ● Complete RIP deficiency (RIPK3^-/-^) | ● ↑ LVEF  ● ↓ LV/BW | ● ↓ RIPK3 | N/A | ● ↑ CD31 (compared with sham)  ● ↓ CD3 (compared with WT)  ● ↓ ROS (compared with WT)  ● ↓ BNP | Chronic MI mediated necroptosis via RIPK3-dependent signaling pathway. | (33) |
| C57BL/6 mice  (22-25 g) | Permanent LAD ligation for 12 weeks | ● ↑ MI size  ● ↑ HW/BW  ● ↑ LVIDd and LVIDs  ● ↑ LVRT and LVCT (within 2 wk)  ● ↑ ET  ● ↑ E/A ratio (8 wk)  ● ↔ E/A ratio (1-4 wk)  ● ↓ LVAW and LVPW  ● ↓ LVFS and LVEF (within 1 wk)  ● ↓ CO  ● ↓ e’/a’ (4 wk) | ● ↑ RIPK1, RIPK3 | N/A | ● ↑ Beclin1 (from 1 d to 12 wk)  ● ↑ LC3 II/I (1-3 d)  ● ↓ LC3 II/I (at 1wk)  ● ↓ p62 (1d)  ● ↑ p62 until 12 wk | ● Chronic MI mediated necroptosis via increased RIPK1, RIPK3 and impaired autophagy flux leading to LV remolding and dysfunction.  ● Impaired autophagy flux mediated necroptosis in chronic MI via decreased Beclin1 expression. | (21) |
|  | **Pre-treatment with**:  ● RIPK3 knockdown | ● ↑ LVEF, LVFS  ● ↑ LVAWd, LVAWs  ● ↑ CO  ● ↓ LVIDd, LVIDs  ● ↓ E/A ratio  ● ↓ LVCT, LVRT  ● ↓ ET | ● ↓ RIPK3 | N/A | N/A |  |  |
|  | ● RIPK3 overexpression | ● ↑ MI size  ● ↑ Cardiac fibrosis  ● ↑ E/A ratio  ● ↑ e’/a’  ● ↑ LVCT, LVRT  ● ↓ LVEF, LVFS  ● ↓ LVAWd, LVAWs  ● ↓ CO | ● ↑ RIPK3 | N/A | N/A |  |  |
|  | ● Beclin1 knockdown | ● ↑ MI size  ● ↑ Cardiac fibrosis  ● ↑ LVID  ● ↑ LVRT, LVCT  ● ↑ E/A ratio  ● ↓ LVEF, LVFS  ● ↓ LVAW  ● ↓ e’/a’  ● ↓ CO | ● ↑ Necrotic cell death  ● ↑ RIPK3 | N/A | ● ↓ Autophagosomes |  |  |
|  | ● Beclin1 overexpression | ● ↑ Cardiac fibrosis  ● ↑ LVEF, LVFS  ● ↑ e’/a’  ● ↓ LVID | ● ↓ Necrotic cell death  ● ↓ RIPK3 | N/A | ● ↑ Autophagosome |  |  |
| C57BL/6J mice  (male, 20-25 g) | Permanent LAD ligation | ● ↑ MI size  ● ↑ Cardiac fibrosis  ● ↑ LVEDD, LVESD  ● ↓ LVFS, LVEF | ● ↑ RIPK1, RIPK3  ● ↑ p-MLKL  ● ↑ RIPK3 mRNA  ● ↑ RIPK 3 | ● ↑ TUNEL positive cells | ● ↓ miR-325-3p expression  ● ↑ LDH, CK, MDA  ● ↓ SOD | Chronic MI mediated necroptosis via increased RIPK1, RIPK3, p-MLKL, RIPK 3 and reduced miR-325-3p leading to LV remolding and dysfunction. | (31) |
|  | **Pre-treatment with**:  ● Antagomir- 325-3p | ● ↑ MI size  ● ↑ Cardiac fibrosis  ● ↑ LVEDD, LVESD  ● ↓ LVFS, LVEF | ● ↑ RIPK1, RIPK3  ● ↑ p-MLKL  ● ↑ RIPK3 mRNA  ● ↑ RIPK3 | ● ↑ TUNEL positive cells | ● ↑ LDH, CK, MDA  ● ↓ SOD |  |  |
|  | ● AgomiR-325-3p | ● ↓ MI size  ● ↓ Cardiac fibrosis  ● ↓ LVEDD, LVESD  ● ↑ LVFS, LVEF | ● ↓ RIPK1, RIPK3  ● ↓ p-MLKL  ● ↓ RIPK3 mRNA  ● ↓ RIPK3 | ● ↓ TUNEL positive cells | ● ↓ LDH, CK, MDA  ● ↑ SOD |  |  |
| Genetically modified mouse models | - Traf2fl/fl-αMHC-Cre | ● ↑ Cardiac fibrosis  ● ↑ LVED, LVES, LVPWd  ● ↓ LVFS | ● ↓ Traf2  ● ↑ HMGB1 | ● ↑ TUNEL positive cells  ● ↑ Bax | ● ↑ PAPR | Decrease in Traf2 expression mediated both necroptosis via RIPK3 signaling and apoptosis leading to LV remodeling, and dysfunction in chronic MI. | (30) |
|  | Traf2fl/fl- βMHC-Cre (Traf2 deficient) | ● ↑ Cardiac fibrosis  ● ↑ HW/BW  ● ↑ LVED, LVES  ● ↓ LVFS | ● ↑ HMGB1 | N/A | N/A |  |  |
|  | RIPK3 ^-/-^+ Traf2fl/fl-αMHC-Cre | ● ↓ Cardiac fibrosis  ● ↓ HW/BW  ● ↓ LVED, LVES  ● ↑ LVFS | ● ↓ HMGB1 | ● ↑ TUNEL positive cells | N/A |  |  |
|  | Permanent LAD ligation for 2 weeks  **Pre-treatment with**:  ● Traf2fl/ +αMHC-Cre | ● ↑ MI size  ● ↑ LVED  ● ↑ IA/AAR  ● ↓ LVFS | ● ↑ Plasma HMGB1 | ● ↑ TUNEL positive cells | ● ↑ cTnI |  |  |
| C57BL/6 mice  (male, 8-10 wk, 18-20 g) | Permanent LAD ligation for 2 weeks  **Pre-treatment with:**  ● Alliin: 100 mg/kg IP for 7 ds | ● ↑ Animals survival  ● ↑ LVFS, LVEF | ● ↓ RIPK1, RIPK3  ● ↓ Traf2 | ● ↓ TUNEL positive cells  ● ↓ CCP3 | ● ↑ Cells viability  ● ↑ Beclin1  ● ↓ LC3 II/I | Alliin improved LV function and survival rate via decreasing necroptosis and apoptosis and increasing autophagy in chronic MI. | (34) |

Bax, BCL2 associated X protein; BCL-2, B-cell lymphoma 2 protein; BNP, B-type natriuretic peptide; cTnI, cardiac troponin I; CD3, cluster of Differentiation 3; CD31, cluster of Differentiation 31; CK creatine kinase; CO, cardiac output; E/A, E wave per A wave ratio; ET, ejection time; HMGB1, high mobility group box 1; HW/BW, heart weight per body weight ratio; LAD, left anterior descending coronary artery; LC3 I, the cytosolic form of microtubule-associated protein 1A/1B-light chain 3; LC3 II, the lipid modified form of microtubule-associated protein 1A/1B-light chain 3; LDH, lactate hydrogenase; LV, left ventricle; LV/BW, left ventricle mass to body weight ratio; LVAWd, left ventricular end-systolic anterior wall thickness during diastole; LVAWs, left ventricular end-systolic anterior wall thickness during systole; LVCT, left ventricular contraction time; LVEDD, left ventricular end-diastolic dimension; LVEF, left ventricular ejection fraction; LVES, left ventricular end systolic volume; LVESD, left ventricular end-systolic dimension; LVFS, left ventricular fractional shortening; LVIDd, left ventricular internal diameter end diastole; LVIDs, left ventricular internal diameter end systole; LVPW, left ventricular posterior wall thickness; LVRT, left ventricular relaxation time; MDA, malondialdehyde; MI, myocardial infarction; N/A, not available; p-MLKL, the phosphorylated form of mixed lineage kinase domain like pseudokinase; p-RIP3, the phosphorylated form of receptor-interacting serine/threonine-protein kinase 3; p62, sequestosome 1; RIPK1, receptor-interacting serine/threonine-protein kinase 1; RIPK3, receptor-interacting serine/threonine-protein kinase 3; RIPK3^-/-^, receptor-interacting serine/threonine-protein kinase 3 gene knockout; ROS, reactive oxygen species; SOD, superoxide dismutase; Traf2, tumor necrosis factor receptor-associated factor 2; TUNEL, terminal deoxynucleotidyl transferase dUTP nick end labeling

**Supplementary Table 3.** Necroptosis in chronic MI: reports from clinical studies.

| **Study model** | **Methods** | **Major finding** | | | **Interpretation** | **Ref.** |
| --- | --- | --- | --- | --- | --- | --- |
|  |  | **Necroptosis** | **Apoptosis** | **Other biochemical parameters** |  |  |
| Patients with CAD | **Plasma/serum** collected from patient with:  ● SCAD (n = 93)  ● UA (n = 153)  ● MI (n = 72) | ● Plasma RIPK3: SCAD > UA > MI > control  ● Plasma RIPK3 > serum RIPK3  ● Plasma RIPK3 levels are associated with CAD (6.00 OR, 95% CI)  ● Plasma RIPK3 levels have positive correlation with CAD severity | N/A | N/A | ● Plasma RIPK3 levels were increased in CAD patients. | (26) |
| Patients with HF | **Peripheral venous blood samples**  (n = 3194) | ● ↑ Plasma RIPK3 (in NYHA III)  ● Plasma RIPK3 of class III, IV > class II and control  ● ↑ CVD-death and cardiac transplantation rate in rs3212247‐CC genotype than TT, TC | N/A | N/A | ● RIPK3 level showed a positive correlation with the severity of HF.  ● Patient carrying rs3212247‐CC genotype showed poor prognosis of HF. | (38) |
| Patients with HF | **LV samples** collected from patient with**:**  ● CAD (n = 6) | ● ↑ RIPK1, RIPK3  ● ↑ pSer^227^-RIPK3  ● ↓ CP8, PCP8  ● ↑ pThr^357^-MLKL, pSer^358^-MLKL | ● Undetectable of p89 fragment (apoptotic marker)  ● ↓ CP7  ● ↔ CP3  ● ↔ Bcl-2  ● ↔ Bax  ● ↔ PARP p25  ● ↑ PARP1 p35 | N/A | ● In CAD and DCM induced HF patients, necroptosis markers were more expressed than apoptosis markers. | (64) |
|  | ● DCM (n = 10) | ● ↑ RIPK1, RIPK3  ● ↑ pSer^227^-RIPK3  ● ↓ CP8  ● ↑ pThr^357^-MLKL, pSer^358^-MLKL | ● Undetectable of p89 fragment (apoptotic marker)  ● ↓ CP7  ● ↓ CP3  ● ↔ Bcl-2  ● ↓ Bax  ● ↔ PARP p25  ● ↑ PARP1 p35 | N/A |  |  |
| Patients with HF | **Myocardial samples** collected from patient with:  ● CAD (n = 10)  ● DCM (n = 12) | ● ↑ RIPK1 positive cells  ● ↑ RIPK3 positive cells | ● ↑ TUNEL positive cell | ● ↑ LC3 II positive cell  ● ↑ NF-κB positive cell | ● HF exhibited both necroptosis and apoptosis. | (63) |

Bax, BCL2 associated X protein; BCL-2, B-cell lymphoma 2 protein; CAD, coronary artery disease; CI, confidence interval; CP7, caspase 7; CP8, caspase 8; CVD, cardiovascular disease; DCM, dilated cardiomyopathy; HF, heart failure; LC3 II, the lipid modified form of microtubule-associated protein 1A/1B-light chain 3; MI, myocardial infarction; N/A, not available; NF-κB, nuclear factor-κB; NYHA, New York Heart Association (NYHA) functional classification; OR, odd ratio; pSer^227^-RIPK3, the phosphorylated form of receptor-interacting serine/threonine-protein kinase 3 at serine 227; pSer^358^-MLKL, the phosphorylated form of mixed lineage kinase domain like pseudokinase at serine 358; pThr^357^-MLKL, the phosphorylated form of mixed lineage kinase domain like pseudokinase at threonine 357; PARP, poly (ADP-ribose) polymerase; RIPK1, receptor-interacting serine/threonine-protein kinase 1; RIPK3, receptor-interacting serine/threonine-protein kinase 3; SCAD, stable coronary artery disease; TUNEL, terminal deoxynucleotidyl transferase dUTP nick end labeling; UA, unstable angina
